# A Network Approach to White Band Disease Challenged Staghorn Coral *Acropora cervicornis* microRNAs and Their Targets

**DOI:** 10.1002/ece3.71351

**Published:** 2025-04-25

**Authors:** Brecia A. Despard, Jason D. Selwyn, Allison N. Shupp, Steven V. Vollmer

**Affiliations:** ^1^ Department of Marine and Environmental Sciences Northeastern University Nahant Massachusetts USA; ^2^ Genomics CORE Laboratory Texas A&M University—Corpus Christi Corpus Christi Texas USA

**Keywords:** cnidarians, disease transmission experiment, innate immunity, microRNA, network analysis, targets

## Abstract

Coral reefs are increasingly threatened by disease outbreaks, yet little is known about the genetic mechanisms underlying disease resistance. Since the 1970s, White Band Disease (WBD) has decimated the Caribbean staghorn coral *Acropora cervicornis* . However, 15% or more of individuals are highly disease‐resistant, and the genes controlling the production of Argonaut proteins, involved in microRNA (miRNA) post‐transcriptional gene silencing, are up‐regulated in WBD‐resistant corals. This suggests that miRNAs may be key regulators of coral immunity. In this study, we conducted an in situ disease transmission experiment with five healthy‐exposed control tanks and five WBD‐exposed tanks, each containing 50 *A. cervicornis* genotypes, sampled over 7 days and then sequenced miRNAs from 12 replicate genotypes, including 12 WBD‐exposed and 12 healthy‐exposed control fragments from two time points. We identified 67 *bona fide* miRNAs in *A. cervicornis* , 3 of which are differentially expressed in disease‐resistant corals. We performed a phylogenetic comparison of miRNAs across cnidarians and found greater conservation of miRNAs in more closely related taxa, including all three differentially expressed miRNAs being conserved in more than one *Acropora* coral. One of the three miRNAs has putative genomic targets involved in the cnidarian innate immunity. In addition, community detection coupled with over‐representation analysis of our miRNA–messenger RNA (mRNA) target network found two key unique *A. cervicornis* miRNAs regulating multiple important immune‐related pathways such as Toll‐like receptor pathway, endocytosis, and apoptosis. These findings highlight how multiple miRNAs may help the coral host maintain immune homeostasis in the presence of environmental stress including disease.

## Graphical abstract

Our study looks at the association between microRNA expression and disease resistance in Caribbean staghorn corals infected with White Band Disease. We find three microRNAs differentially expressed due to disease resistance with putative targets involved in innate immunity. We also provide an updated phylogenetic analysis of conserved microRNAs within the cnidarian phylum.

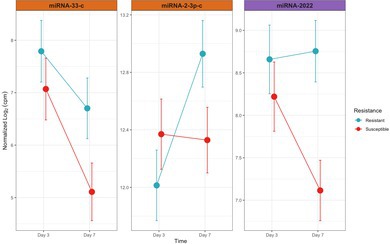

## 1 Introduction

MicroRNAs (miRNAs) are a class of small non‐coding RNAs that can silence or inhibit gene expression through sequence complementarity to their targets (Aalto and Pasquinelli 2012; Ladomery et al. 2011) by binding to Argonaute (AGO) protein and creating an RNA‐induced silencing complex (RISC) (Fabian and Sonenberg 2012; Frédérick and Simard 2022). They regulate diverse biological processes ranging from development to cell signaling, homeostasis, and apoptosis (Ebert and Sharp 2012). To date, the majority of research on miRNAs has been done in bilaterian metazoans and land plants (Li and Hui 2023). Bilaterians are a large clade of animals characterized by bilateral symmetry, and this clade of animals does not include the basal metazoans including phyla Cnidaria, Porifera, or Placozoa (Freeman 2009). Over 30 phylogenetically conserved families of miRNAs have been identified in bilaterians (Prochnik et al. 2007); only a handful of conserved miRNAs have been identified in plants (Jones‐Rhoades 2012), and no shared miRNAs between bilaterians and plants (Wang et al. 2024) have been discovered, possibly due to high miRNA turnover rates in plants and non‐bilaterian animals (Grimson et al. 2008; Moran et al. 2014, 2017).

Cnidarian miRNAs have characteristics of both plant and bilaterian miRNAs from biogenesis (Li and Hui 2023; Moran et al. 2013) to target regulation (Fridrich et al. 2023; Moran et al. 2014). Pioneering research in the sea anemone *Nematostella vectensis* suggests that cnidarian miRNAs target messenger RNAs (mRNAs) with extended complementarity that frequently cause transcript cleavage, a mechanism previously thought to occur only in plants (Moran et al. 2014). Cnidarian miRNAs also have validated targets in both the coding domain sequence (CDS) and 3′ untranslated region (3′ UTR) of mRNA transcripts (Baumgarten et al. 2018; Moran et al. 2014; Praher et al. 2021), combining mechanisms of both plant and bilaterian miRNAs, respectively (Hausser et al. 2013; Lee and Shin 2012). In addition, the miRNA pathway components present in cnidarians contain genes involved in both bilaterian miRNA biogenesis including Dicer, Drosha, Pasha, and GW182 and plant miRNA biogenesis including HEN1 (HUA Enhancer 1), HYL1 (Hyponastic Leaves 1) and Serrate (Moran et al. 2013; Shang et al. 2023; Tripathi et al. 2022). Praher et al. (2021) conducted a comprehensive analysis of conserved vs. lineage‐specific miRNAs in cnidarians that included 10 cnidarian species. They found one miRNA (miRNA‐100) shared with bilaterians, two miRNAs conserved in all Cnidaria and six conserved miRNAs occur within Anthozoa (Praher et al. 2021).

While cnidarian miRNA gene regulation may share plant and bilaterian characteristics, little is known about how cnidarians use miRNAs to regulate gene expression. To date, only two studies have investigated the role of cnidarian miRNAs in experimentally challenged species. Gajigan and Conaco (2017) researched the stony coral, *Acropora digitifera* miRNA response to heat stress, and Baumgarten et al. (2018) looked at miRNA expression in the symbiotic anemone, *Exaiptasia pallida*, and its response to algal endosymbiont infection. Both papers document an indirect role of differentially expressed miRNAs in stress response, where there were tens to hundreds of putative targets per miRNA, with only five and eight targets contributing to the onset and maintenance of endosymbiosis and to heat tolerance. This led to the conclusion that differentially expressed miRNAs regulate the expression of multiple target genes during stress (Baumgarten et al. 2018; Gajigan and Conaco 2017).

Cnidarian miRNA response to disease exposure or infection has yet to be studied. Cnidarians contain only an innate immune system and therefore have a generalized approach to pathogen exposure involving multiple pathogen recognition receptors and downstream signaling processes (Bosch and Rosenstiel 2015). *A. cervicornis* genotypes display strong phenotypic variation in disease resistance (Vollmer and Kline 2008) and mount a vigorous immune response to White Band Disease (WBD) infection (Libro et al. 2013) in addition to having 10 genomic regions highly associated with disease resistance that contain four genes with protein‐coding changes involved in coral immunity and pathogen detection (Vollmer et al. 2023).

The staghorn coral, WBD host–pathogen system, is an ideal system to investigate the potential role of miRNAs in staghorn coral WBD resistance because it is a highly transmissible disease (Vollmer and Kline 2008) that can be experimentally transmitted in tanks to replicate coral genotypes (Selwyn et al. 2024) across a range of disease resistances (Muller et al. 2018; Vollmer and Kline 2008). WBD caused unprecedented Caribbean‐wide die‐offs in two *Acropora* species, *A. cervicornis* and *Acropora palmata* , since the late 1970s (Aronson and Precht 2001) and resulted in their listing as endangered under the Endangered Species Act. About 15% of *A. cervicornis* are resistant to WBD (Vollmer and Kline 2008), and AGO proteins were shown to be up‐regulated in WBD‐resistant *A. cervicornis* fragments regardless of disease exposure (Libro and Vollmer 2016). AGO is the primary protein in the RISC complex that causes post‐transcriptional gene regulation via miRNAs (Fridrich et al. 2020; Kobayashi and Tomari 2016) suggesting miRNAs may play a key role in coral disease resistance. Some AGO proteins in Cnidaria also bind to short‐interfering RNAs (siRNAs), another class of small RNAs, and contribute to antiviral defense (Li and Hui 2023).

In this study, we characterized miRNA diversity and expression across 12 *A. cervicornis* genotypes exposed to disease in a transmission experiment. We then compared the miRNA diversity of *A. cervicornis* to known cnidarian miRNA diversity from 15 other species, focusing on unique and shared miRNAs within and among the scleractinians, anthozoans, and cnidarians. To decipher *A. cervicornis* miRNAs' roles in disease resistance, we used network and differential expression analyses to understand the interactions between miRNAs and their putative mRNA targets, focusing on key immune regulators.

## 2 Methods

### 2.1 miRNA Sequencing

miRNAs were sequenced from total RNA samples of 48 *A. cervicornis* fragments used in a tank‐based disease transmission experiment conducted at the Florida Keys Marine Laboratory in June 2021. Ten replicate fragments from each putative genotype were spread across one of ten 18‐l recirculating tanks at ambient seawater temperatures, a flow‐thru seawater system. Each fragment was experimentally lesioned with a waterpik to facilitate transmission (Gignoux‐Wolfsohn et al. 2012). Five tanks were exposed to 50 mL of disease slurry produced from 10 WBD‐infected coral fragments, and five tanks were exposed to 50 mL of healthy slurry from 10 healthy fragments. Slurries were produced by water‐piking disease or healthy coral tissue off the sampled corals in filtered seawater (FSW). The slurry doses were normalized to a standard ocular density of 0.6 at 600 nm. Exposed coral tanks were censused for disease twice daily, and disease coral fragments were pulled from tanks at the first signs of disease to prevent amplifying pathogen spread within each tank (Vollmer et al. 2023). Coral fragments were sampled at two time points (3 and 7 days) after exposure to the healthy or disease slurries. If a coral fragment showed disease symptoms before 7 days post‐exposure, it was sampled and removed to prevent disease amplification. A genotype was termed resistant if it did not contract disease in four out of the five disease‐exposed tanks, and susceptible corals contracted disease in all five disease‐exposed tanks. Tissue samples were preserved in TRIzol reagent (Invitrogen) and placed in a − 80°C freezer until they were extracted.

Total RNA was extracted using with Zymo Research Directzol‐96 RNA kit and small RNA libraries were prepared using QIAseq miRNA Library Prep Kit for Illumina NGS Systems (Qiagen) both following the manufacturer's protocol and sequenced on two 75‐bp single‐end Illumina NextSeq500 lanes. Six resistant and six susceptible genotypes were sequenced from each exposure treatment totaling 12 *A. cervicornis* genotypes with each genotype sequenced at days 3 and 7 after exposure to healthy or disease slurries (12 genotypes × 2 exposures × 2 times = 48 samples). fastp (Chen et al. 2018) was used to remove adapters and barcodes, filter low‐quality sequences (PHRED < 30), trim sequences shorter than 15 bp, and remove PCR artifacts. Contaminants were removed with FASTQ_SCREEN (Wingett and Andrews 2018) by mapping reads against a suite of potential contaminant genomes (e.g., human, viral, bacterial) as well as 13 available genomes of *Symbiodiniaceae* (Table 1) and removing reads that had hits in any potential contaminant genome. A two‐way ANOVA was done to validate no significant difference in read depth for exposure and resistance experimental variables and the interaction between the two.

**TABLE 1:** National Center for Biotechnology Information accession numbers and citations for *Symbiodinium* reference genomes, which reads were mapped against to identify *Symbiodinium* composition within each sample. Species names have been adjusted *sensu* LaJeunesse et al. (2018).

Species	Clade	Accession #	Citations
*Symbiodinium microadriaticum*	A	GCA_001939145	Aranda et al. (2016)
*Symbiodinium* sp. clade A Y106	A	GCA_003297005	Shoguchi et al. (2018)
*Cladocopium* sp. clade C Y103	C	GCA_003297045	Shoguchi et al. (2018)
*Fugacium kawagutii*	F	GCA_009767595	Lin et al. (2015)
*Symbiodinium microadriaticum*	A	GCA_018327485	Yoshioka et al. (2021)
*Symbiodinium natans*	A	GCA_905221605	González‐Pech et al. (2021)
*Symbiodinium* sp. CCMP2592	A	GCA_905221615	González‐Pech et al. (2021)
*Symbiodinium* sp. KB8	A	GCA_905221625	González‐Pech et al. (2021)
*Symbiodinium* sp. CCMP2456	A	GCA_905221635	González‐Pech et al. (2021)
*Symbiodinium pilosum*	A	GCA_905231905	González‐Pech et al. (2021)
*Symbiodinium necroappetens*	A	GCA_905231915	González‐Pech et al. (2021)
*Symbiodinium microadriaticum*	A	GCA_905231925	González‐Pech et al. (2021)
*Breviolum minutum* Mf 1.05b.01	B	GCA_000507305	Shoguchi et al. (2013)

### 2.2 miRNA Annotation

miRNAs were identified using miRDeep2 (Friedländer et al. 2012) with the recently published *A. cervicornis* genome as a reference (Selwyn and Vollmer 2023). Redundant sequences were collapsed using collapse_reads.pl package from miRDeep2 (Friedländer et al. 2012). Before running the miRDeep2 core algorithm (miRDeep2.pl), small RNA reads were mapped to non‐coding RNA regions such as rRNAs or tRNAs with bowtie2 (Langmead and Salzberg 2012) from the annotated genome of *A. cervicornis* (Selwyn and Vollmer 2023) and removed. miRDeep2.pl was run using the default settings and candidate miRNAs were considered *bona fide* if they met six criteria. Criteria specific to the miRDeep2 program included (1) having a miRDeep2 score greater than 10, (2) a significant RNAfold *p*‐value, and (3) a minimum of 10 reads total per miRNA. In addition, miRNAs were manually filtered with criteria summarized by Fromm et al. (2015), including (4) a 2‐nt overhang on the 3' end of precursor miRNA, (5) 5' consistency of mature miRNA strand (90% of reads starting with the same nucleotide) and (6) at least 16‐nt complementarity between the mature and star strands.

To examine phylogenetic conservation of cnidarian miRNAs, we created a reference library of mature miRNA sequences from *A. cervicornis* and 16 additional cnidarian species. These species included one hydroid—*Hydra vulgaris* formerly *H*ydra *magnipapillata* (Krishna et al. 2013; Macias‐Muñoz et al. 2019), three scyphozoans (jellies)— *Aurelia aurita* , *Sanderia malayensis* , *Rhopilema esculentum* (Nong et al. 2020), one octocoral— *Heliopora coerulea* (Ip et al. 2023), and 10 anthozoans including seven sea anemones— *Edwardsiella carnea* , *Scolanthus callimorphus*, *Metridium senile* , *Anemonia viridis* (Praher et al. 2021), *Nematostella vectensis* (Fridrich et al. 2020), *Edwardsia elegans* (Rutlekowski et al. 2025) and *Exaiptasia pallida* (Baumgarten et al. 2018), and four stony corals— *Acropora millepora* (Praher et al. 2021), *Acropora digitifera* (Gajigan and Conaco 2017), *Stylophora pistillata* (Liew et al. 2014), and *Cataphyllia jardinei* (Yu et al. 2022).

We considered a mature miRNA sequence homologous with another cnidarian miRNA if it met the criteria presented in Wheeler et al. (2009), including (1) sequence length matches within two nucleotides, (2) positions two through seven on the mature strand are exact matches, and (3) three or fewer mismatches in the remainder of the mature sequence. The only exception is miRNA‐100, which has a 1‐nt seed shift in the bilaterian mature miRNA sequence, which is well documented in miRNA literature (Grimson et al. 2008). For a miRNA to be considered conserved at any branch of the cnidarian phylogenetic tree, it had to be present in > 50% of the species contained within the branch and in more deeply conserved branches (i.e., Anthozoa and Cnidaria) the miRNAs needed to be present in at least one anemone and one stony coral. Conserved miRNA names with corresponding consensus sequences are presented as the most common name used in the previous literature with species prefix(es) replaced with “miRNA” (Table 2). For names not in a database (miRBase or mirGeneDB) the suffix “c” was added to stand for cnidarian. All unique *A. cervicornis* miRNAs begin with the prefix “Acerv_scaffold.” The common name used along with the paper naming source from which we retrieved these common names is presented in Table 2. miRNAs were categorized into three classes—unique, acroporid, and cnidarian—where acroporid miRNAs are conserved with one or both of *A. millepora* and *A. digitifera* and cnidarian miRNAs are conserved outside of the Acroporidae family.

**TABLE 2:** List of conserved miRNA names (not including ones in just scyphozoans), the miRNA class, miRNA consensus sequence, the literature source from which the name came from, and the original name from that source. All previous name prefixes replaced with “miRNA” but kept the numbers trailing the same. For names not in a database (miRBase or mirGeneDB), the suffix “c” was added to stand for cnidarian.

miRNA name	miRNA class	miRNA consensus sequence	Source	miRNA original name
miRNA‐100	Cnidarian	ucccguagauccgaacuugugg	Praher et al. (2021)	miRNA‐100
miRNA‐2022	Cnidarian	uuugcuaguugcuuuugucccgu	Praher et al. (2021)	miRNA‐2022
miRNA‐2030	Cnidarian	uagcauaacauuguaagagauc	Praher et al. (2021)	miRNA‐2030
miRNA‐2023	Cnidarian	aaagaaguacaagugguaggg	Praher et al. (2021)	miRNA‐2023
miRNA‐2025	Cnidarian	auuuuuagcccgcggaaguugc	Praher et al. (2021)	miRNA‐2025
miRNA‐2036	Cnidarian	uauauuguacgacucucaucgugu	Praher et al. (2021)	miRNA‐2036
miRNA‐2037	Cnidarian	ugugauuggagacuuuuaucgu	Praher et al. (2021)	miRNA‐2037
miRNA‐2050	Cnidarian	uuugauugcugugaucugguua	Praher et al. (2021)	miRNA‐2050
miRNA‐9425	Cnidarian	aagaacacccaaaauagcugagga	Praher et al. (2021)	miRNA‐9425
miRNA‐14‐c	Cnidarian—Scleractinia	caauguuucggcuuguucccg	Liew et al. (2014)	spi‐L‐miR‐temp‐14
miRNA‐2026	Cnidarian—Actiniaria	aauuucaaauauccacugauug	Fridrich et al. (2020)	mse‐nve‐F‐miR‐2026
miRNA‐12426‐c	Cnidarian—Actiniaria	uaagcucggagcaugcuuucaca	Baumgarten et al. (2018)	mse‐apa‐B‐miR‐12426
miRNA‐12448‐c	Cnidarian—Actiniaria	uauaagucuaggcugguuaa	Baumgarten et al. (2018)	mse‐apa‐B‐miR‐12448
miRNA‐2‐c	Acroporid	uacaaaaacaagaugagugcagg	Praher et al. (2021)	adi‐ami‐miR‐P‐novel‐2
miRNA‐4‐c	Acroporid	aaaaaugucgguugcuuaagcu	Praher et al. (2021)	adi‐ami‐miR‐P‐novel‐4
miRNA‐10‐c	Acroporid	ucggacaccuguaauuggaua	Gajigan and Conaco (2017)	ami‐Adi‐MiR‐G‐Novel‐10‐3p
miRNA‐13‐c	Acroporid	uaaggaggaagcaugauacgua	Praher et al. (2021)	adi‐ami‐miR‐P‐novel‐13
miRNA 29‐c	Acroporid	uuauggauaucaguuuucuuuc	Praher et al. (2021)	adi‐ami‐miR‐P‐novel‐29
miRNA‐1‐3p‐c	Acroporid	uuaacgaguagauaaaugaagag	Praher et al. (2021)	adi‐miR‐P‐novel‐1‐3p
miRNA‐3‐3p‐c	Acroporid	uguucucugcaauagccugccuc	Praher et al. (2021)	adi‐ami‐miR‐P‐novel‐3‐3p
miRNA‐5‐3p‐c	Acroporid	caagugagagaagguuagugugg	Gajigan and Conaco (2017)	ami‐Adi‐MiR‐G‐Novel‐5‐3p
miRNA‐24‐3p‐c	Acroporid	uauugaaauaagauuggauaua	Praher et al. (2021)	adi‐ami‐miR‐P‐novel‐24‐3p
miRNA‐7‐c	Acroporid	ucauaacagugaggaccauucu	Praher et al. (2021)	adi‐ami‐miR‐P‐novel‐7
miRNA‐2‐3p‐c	Acroporid	ucuggcaguauguuauuuuuccaau	Praher et al. (2021)	adi‐miR‐P‐novel‐2‐3p
miRNA‐4‐3p‐c	Acroporid	uuuuugugauguucgucaauau	Praher et al. (2021)	adi‐miR‐P‐novel‐4‐3p
miRNA‐5‐c	Acroporid	uuucaaauuaggaagggagguguu	Praher et al. (2021)	ami‐miR‐P‐novel‐5
miRNA‐19‐c	Acroporid	ucaugggcuauugacccguagc	Praher et al. (2021)	avi‐ami‐miR‐P‐novel‐19
miRNA‐33‐c	Acroporid	acgcuaggaagggaugccggga	Praher et al. (2021)	avi‐ami‐miR‐P‐novel‐33
miRNA‐7‐3p‐c	Acroporid	uugaguuuucaacuauuggauu	Praher et al. (2021)	adi‐miR‐P‐novel‐7‐3p
miRNA‐8‐3p‐c	Acroporid	acugcagcuaaauacuccgcugc	Praher et al. (2021)	ami‐miR‐P‐novel‐8‐3p
miRNA‐17‐3p‐c	Acroporid	uaaagcuuuugugaagaaacacg	Praher et al. (2021)	ami‐miR‐P‐novel‐17‐3p
miRNA‐6‐c	Pacific Acroporid	ucugccaaucgucagacaaacua	Praher et al. (2021)	adi‐ami‐miR‐P‐novel‐6
miRNA‐16‐c	Pacific Acroporid	ugguguaccuguaguuuauuuu	Praher et al. (2021)	adi‐ami‐miR‐P‐novel‐16
miRNA‐27‐c	Pacific Acroporid	uagcgagaaaggggcugaacauuu	Praher et al. (2021)	adi‐ami‐miR‐P‐novel‐27
miRNA‐9‐3p‐c	Pacific Acroporid	aaaaauuucguuucagggc	Praher et al. (2021)	adi‐ami‐miR‐P‐novel‐9‐3p
miRNA‐10‐3p	Pacific Acroporid	uuugaaaaugauaugccacaug	Praher et al. (2021)	ami‐miR‐P‐novel‐10‐3p
miRNA‐23‐3p	Pacific Acroporid	uaugggucgacagucgacgguc	Praher et al. (2021)	adi‐ami‐miR‐P‐novel‐23‐3p

### 2.3 miRNA Differential Expression and Their Predicted Targets

Read counts for each miRNA from the 48 samples were computed from miRNA structure output files by miRDeep2 and normalized for variable sequencing depth using the trimmed mean of M values (TMM) method implemented in edger (Robinson et al. 2010). Precision weights were calculated with variancePartition (Hoffman and Schadt 2016). Normalized read counts were converted to log_2_ counts per million and used in weighted linear mixed effects models using LME4 (Bates et al. 2003) with fixed effects of sampling time (day 3 vs. day 7), exposure (healthy vs. diseased), and resistance (resistant vs. susceptible). To control for repeated measurements and potential tank effects, we included random effects of genotype, tank, and fragment ID, a unique fragment identifier. The significance of fixed effects was assessed using F‐tests and the Kenward–Rogers method of calculating denominator degrees of freedom (Kenward and Roger 1997). *p*‐values were adjusted to control for false discovery rate (FDR) (Benjamini and Hochberg 1995) to account for multiple comparisons, with a *p*‐value ≤ 0.05 considered to be differentially expressed.

### 2.4 miRNA Target Prediction and Annotation

Putative targets of *bona fide* *A. cervicornis* miRNAs were predicted using the pita algorithm (Kertesz et al. 2007). pita requires both the *A. cervicornis* mRNA regions of interest and *A. cervicornis* mature miRNA fasta file to retrieve putative targets, using validated genes in the *A. cervicornis* genome (Selwyn and Vollmer 2023) as input with default settings. pita starts by scanning the mRNA regions of interest, including potential miRNA binding sites in the CDS and 3′ UTR regions, and then scores each site. 3′ UTR annotations were created with TransDecoder (Haas et al. 2013) and added to the previously annotated *A. cervicornis* transcriptome which already contained locations of CDS regions (Selwyn and Vollmer 2023). For stringency, only targets with a seven or eight nucleotide seed match containing no mismatches, and one G: U wobble pair were used (Kertesz et al. 2007). Each site was scored by calculating ΔΔG, which is the difference between the energy gained with miRNA: mRNA binding (ΔG_duplex_) and the energy required to bind by unpairing the target‐site nucleotides (ΔG_open_). Targets with a ΔΔG ≤ −10 were retained for target analysis. To reduce the number of false‐positive target matches and the knowledge of extended seed matches within Cnidaria (Moran et al. 2014), pita targets were further filtered by extending the seed match to 13 bases with two mismatches allowed and bases 10 and/or 11 had to be complimentary to the target strand as that is shown to be the cleavage site of cnidarian miRNAs (Moran et al. 2014).

miRNA predicted gene targets in the *A. cervicornis* genome were either unannotated, had a Swiss‐Prot annotation, or a Kyoto Encyclopedia of Genes and Genomes (KEGG) orthology (KO) term annotation. All KO annotations also have Swiss‐Prot annotations, and therefore annotation numbers presented are subsets of each other. The *A. cervicornis* genome has 15,091 distinct orthogroups that were functionally annotated using BLAST (*e*‐value < 1 × 10^−6^) against the Swiss‐Prot curated portion of the UniProt database (Selwyn and Vollmer 2023; The UniProt Consortium 2023). Consensus KEGG gene annotations were matched to orthogroups based on sequence similarity, and KEGG ortholog membership within KEGG pathways was identified using keggrest (Tenenbaum and Volkening 2023). Putative miRNA targets that mapped to locations in the genome that coded for genes of KEGG orthology terms in the major category Human Diseases were excluded from any target analysis.

### 2.5 miRNA–mRNA Target Network Analysis

An unweighted bipartite network was created for miRNAs and their respective predicted mRNA targets using the extended 13‐seed filtered targets. All network indices were computed using bipartite (Dormann et al. 2008). Degree, betweenness, and degree of specialization (d') (Blüthgen et al. 2006) were all used to evaluate each miRNA's relevant contribution to the gene regulation network and visualized in gephi (Bastian et al. 2009). The network was then filtered to include only targets of at least two miRNAs to perform community detection. Community detection was done via Barber (2007) modularity implemented with the bipmod algorithm (Treviño et al. 2015), which is optimized for unweighted bipartite networks (Thébault 2013).

We analyzed our network focusing on comparing differences between our three miRNA classes (i.e., unique, acroporid, and cnidarian) to try and identify systematic differences between deeply conserved miRNAs compared to unique miRNAs. To do so, we ran several one‐way ANOVAs on our centrality measures, log‐transformed target counts, and target counts for each of our annotation strategies (i.e., total number of targets, Swiss‐Prot annotations, and KO annotations). To understand what functions are highlighted in our miRNA targets, we ran two types of over‐representation analysis using Fisher's exact tests. First, we tested to see if any KEGG pathways occurred significantly more in certain miRNA classes and if there was an over‐representation of KEGG pathways in our network modules. *p*‐Values were FDR‐corrected, and an adjusted *p*‐value of ≤ 0.05 was considered significant for all statistical tests including ANOVAs and over‐representation analysis.

Lastly, given past evidence of cnidarian miRNAs behaving like plant miRNAs with high complementarity to their targets (Moran et al. 2014), we wanted to test another aspect of plant miRNA target recognition by looking to see if cnidarian miRNAs preferentially target CDS regions over 3′ UTRs with only one miRNA instead of multiple (Afonso‐Grunz and Müller 2015; Brodersen and Voinnet 2009; Dai et al. 2011). To do this we ran a binomial regression to identify what region (either CDS or 3′ UTR) is more likely to be a target of one miRNA. We also tested to see if there was a difference in the proportion of targets located in either of these regions with the chi‐square test. All network and statistical analyses were done in R v4.2.1 (R Core Team 2022).

## 3 Results

### 3.1 miRNA Sequencing and Classification of *A. cervicornis* miRNAs

**FIGURE 1:**
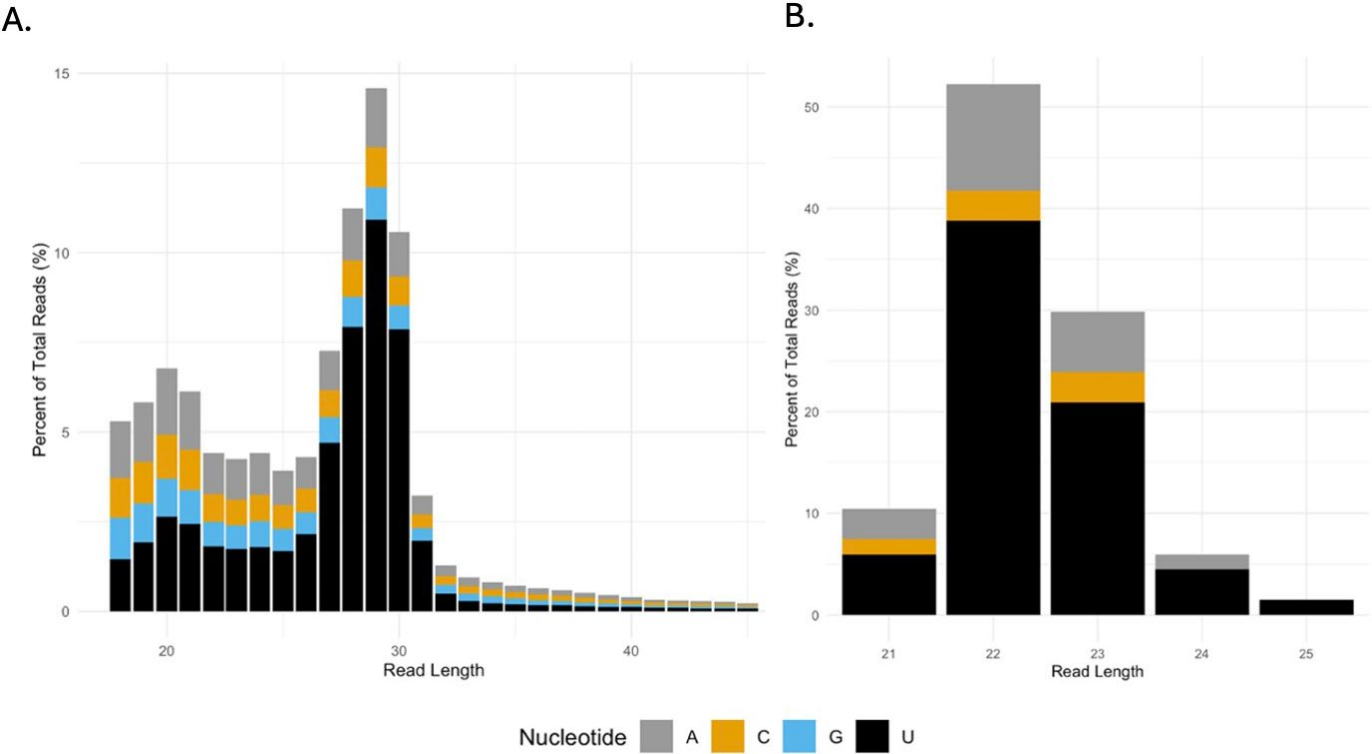
(A) Percentage of the total reads for each starting nucleotide in the collapsed and non‐redundant small RNA sequences. (B) Starting nucleotide percentage for the 67 *A. cervicornis* *bona fide* miRNAs.

Small RNA sequencing data was obtained from 48 *A. cervicornis* samples from 12 genotypes across two time points and two exposures (disease and healthy control tanks) and produced a total of 385,871,359 quality filtered and decontaminated reads, and of those 370,238,577 mapped to the genome (~96.5% ± 0.44% SE). There was an even distribution of read depth for the experimental variables of exposure (*F*_1,44_ = 0.468, *p* = 0.98) and resistance (*F*_1,44_ = 1.666, *p* = 0.204) treatments, and no significant interaction between the two treatments (*F*_1,44_ = 1.769, *p* = 0.19) (Table 3). Collapsed, non‐redundant reads totaled 74,718,997 with a length distribution ranging from 18 to 45 nucleotides (nt) long, with a small peak at 20‐nt corresponding to the expected size range of mature miRNAs, and a larger peak at 29‐nt that may correspond to another class of small RNAs known as piwi‐interacting RNAs (piRNAs). All reads showed a strong affinity toward 5′ uracil, which is a characteristic of both miRNAs and piRNAs (Kutter and Svoboda 2008; Figure 1A). Interestingly, piRNAs are the most abundant type of small RNA in Cnidaria studied in both *Hydra* and *Nematostella* (Calcino et al. 2018; Juliano et al. 2014; Li and Hui 2023; Praher et al. 2017).

**TABLE 3:** Tank experiment sequencing summary showing the day of sampling and mean sequencing depth ± SE for experimental variable exposure (healthy or diseased) and resistance (resistant or susceptible) coral fragments and average across time.

Time	Reads			
	Healthy exposed – Resistant ± SE	Healthy exposed – Susceptible ± SE	Disease exposed—Resistant ± SE	Disease exposed—Susceptible ± SE
Day 3	5,260,270 ± 950,763	4,164,175 ± 732,725	6,136,922 ± 670,781	6,152,946 ± 1,593,072
Day 7	6,964,012 ± 906,426	18,488,068 ± 4,898,790	8,658,712 ± 1,353,629	8,486,788 ± 4,073,311
Total	6,112,141 ± 676,863	11,326,121 ± 3,199,857	7,397,817 ± 814,388	7,319,867 ± 2,114,583

Abbreviation: SE, standard error.

**FIGURE 2:**
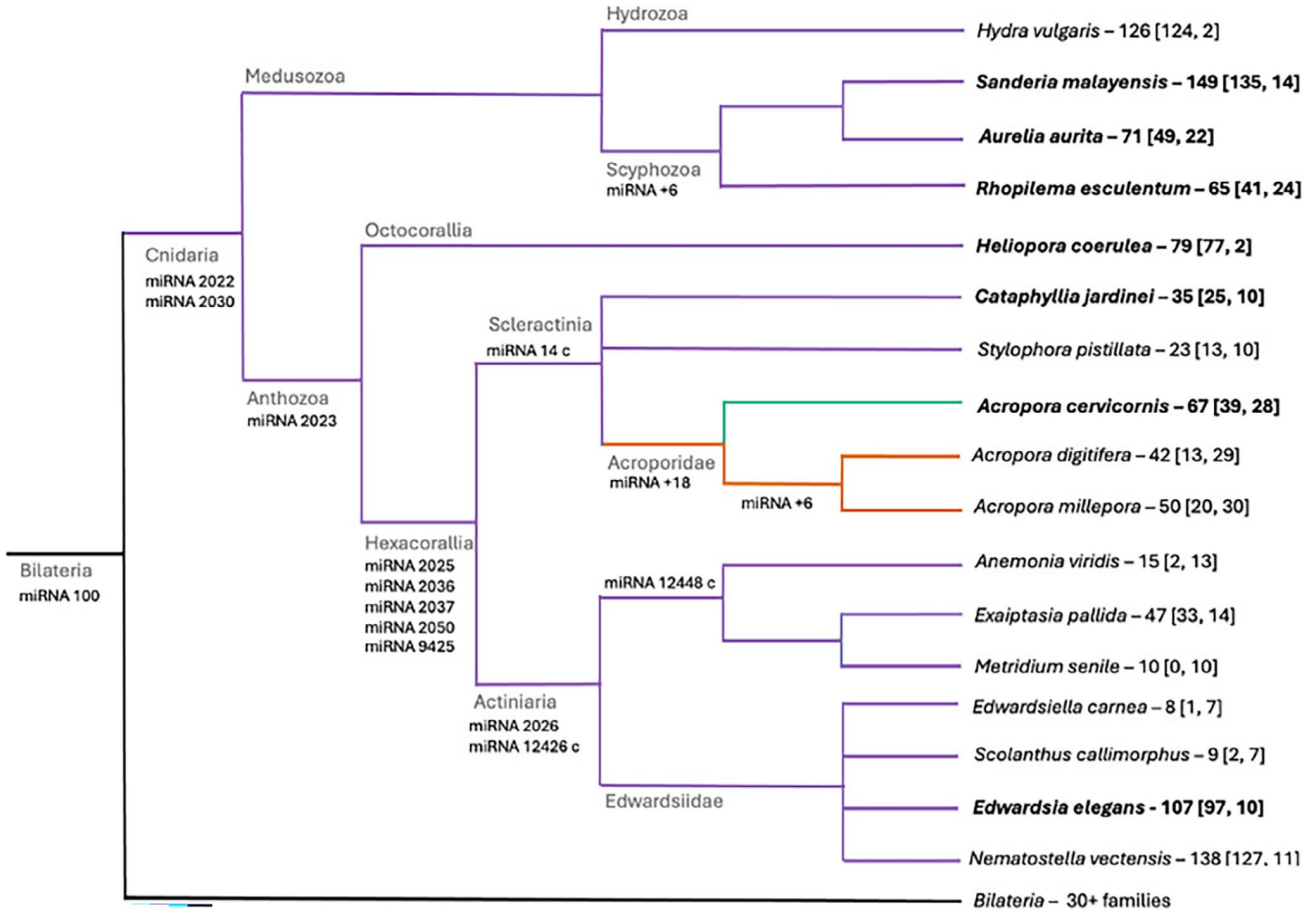
Shared miRNAs mapped on the Cnidaria phylogenetic tree. Species names are followed by their total number of miRNAs, with the number unique and conserved miRNAs in brackets [unique, conserved]. The bolded species names are newly added species from the most recent study by Praher et al. (2021). Colors indicate whether the miRNAs were defined as cnidarian (purple), acroporid (orange), or unique (green). Shared miRNAs are listed under branch names apart from the Acroporidae family and Scyphozoa. The names of acroporid shared miRNAs can be found in Table 2. The six shared miRNAs in Scyphozoa represented have no common naming schematic.

We identified 67 *bona fide* *A. cervicornis* miRNAs. mirDeep2 analysis predicted 580 putative miRNAs, 380 of which had a miRDeep2 score greater than 10, a significant RNAfold *p*‐value, and a minimum of 10 reads total per miRNA. From those, 58 candidate miRNAs met the criteria of *bona fide* miRNAs outlined by Fromm et al. (2015) by containing a 2‐nt overhang on the 3′ end of precursor miRNA, 5′ consistency of mature miRNA strand (90% of reads starting with the same nucleotide) and at least 16‐nt complementarity between the mature and star strands. Nine of the 67 *bona fide* miRNAs failed one filtering criterion but were retained because they occurred in at least two other cnidarian species in our reference library of mature miRNAs. All the predicted *bona fide* miRNAs fell within a length of 21–25 nucleotides with a preference towards uracil as the starting nucleotide, and most miRNAs (> 50%) are 22‐nt in length, as expected (Ranganathan and Sivasankar 2014; Figure 1B). Out of the 67 *bona fide A. cervicornis* miRNAs, 28 are conserved with one or more of the 15 cnidarians in the expanded miRNA dataset validated by Fromm et al. (2015) criteria, while 39 *bona fide* miRNAs are unique to *A. cervicornis* (Figure 2).

### 3.2 Phylogenetic Conservation of Cnidarian miRNAs

Our phylogenetic analysis of cnidarian miRNAs from 17 taxa included three of the four cnidarian classes—three scyphozoans, one hydrozoan, and 13 anthozoans (Figure 2). miRNA‐100 is the only miRNA conserved with bilaterians but is lost in both Scyphozoa and Hydrozoa classes. There are two miRNAs conserved in all Cnidaria, miRNA‐2030 and miRNA‐2022, with miRNA‐2030 present in all 16 species and miRNA‐2022 missing from two species, *Heliopora coerulea* (octocoral) and *Scolanthus callimorphus* (anemone). Even though miRNA‐2030 is present in all species, opposite arms of this miRNA are dominantly processed in Anthozoa and Medusozoa; therefore, the mature sequence is different in the cnidarian lineages (Krishna et al. 2013). Prior literature places six conserved miRNAs within Anthozoa (Praher et al. 2021); however, the addition of octocoral, *H. coerulea* , which splits class Anthozoa into subclasses Octocorallia and Hexacorallia, indicates only miRNA‐2023 is conserved within Anthozoa and miRNA‐2025, miRNA‐2036, miRNA‐2037, miRNA‐2050, and miRNA‐9425 are conserved within Hexacorallia (Figure 2).

There were 12 species total within anthozoan subclass Hexacorallia, which consists of both stony corals (Scleractinia—five species) and anemones (Actiniaria—seven species). Overall, there was relatively low conservation of miRNAs within the anemones (only three miRNAs), but 25 conserved miRNAs within stony corals. Only one (miRNA‐14‐c) out of the 25 conserved miRNAs in stony corals was shared outside the acroporids. In the remaining 24 shared acroporid miRNAs, the Pacific *Acropora* species *A. millepora* and *A. digitifera* share six miRNAs, whereas *A. cervicornis* —the only Caribbean *Acropora* species examined—shares 18 miRNAs with at least one of the Pacific *Acropora* species (Figure 2).

### 3.3 *Acropora cervicornis* Differential Expression of miRNAs and Predicted mRNA Targets

**FIGURE 3:**
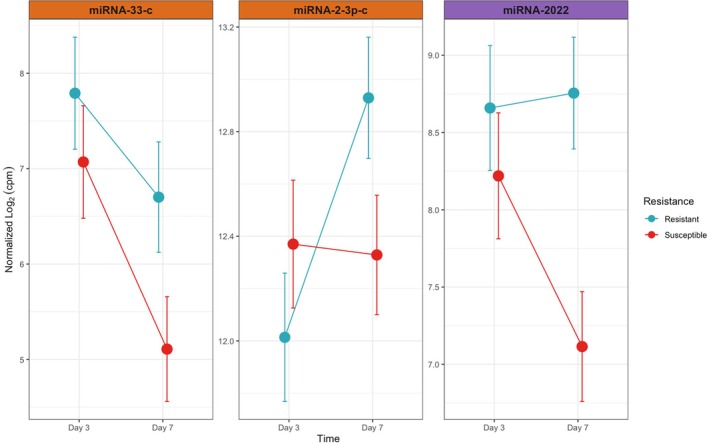
Significantly differentially expressed miRNAs due to resistance treatment variable. Normalized log_2_ counts per million are shown for days 3 and 7 of the transmission experiment. Error bars indicate 95% confidence intervals. The color header indicates either the miRNA is an acroporid miRNA class (orange) or cnidarian miRNA class (purple).

We identified 45 miRNAs significantly differentially expressed through time; three of those were also differentially expressed due to disease resistance or the interaction between time and disease resistance (Figure 3 and Table 4). All three differentially expressed miRNAs involved with disease resistance had a 13‐seed match. No miRNAs were differentially expressed due to exposure. Cnidarian miRNA‐2022 and acroporid miRNA‐2‐3p‐c were both differentially expressed for the interaction between time and resistance, with miRNA abundance showing opposite trends for disease resistance. miRNA‐2022 was up‐regulated in resistant corals, while susceptible genotypes had consistent expression over time, whereas miRNA‐2‐3p‐c was down‐regulated in susceptible genotypes and consistently expressed over time in resistant genotypes (Figure 3). Acroporid miRNA‐33‐c was significantly differentially expressed for disease resistance alone, where both resistant and susceptible corals were down‐regulated over time, with miRNA abundance higher in resistant corals at both time points. A portion of the predicted target mRNAs of all three differentially expressed miRNAs associated with disease resistance are involved in innate immunity, but only one, miRNA‐2022, is suggested to be involved in cnidarian innate immunity. miRNA‐2022 had 19 total putative targets with KO annotations, and two are associated with cnidarian innate immunity, including tumor necrosis factor (TNF) receptor‐associated factors 4 and 6 (TRAF4 and TRAF6), and one associated with higher metazoan immunity, E3 ubiquitin‐protein ligase synoviolin. miRNA‐2‐3p‐c had 14 putative targets with KO annotations, with gamma‐aminobutyric acid (GABA) type A receptor‐associated protein (GABARAP) and mitogen‐activated protein kinase 12 (MAP3K12) binding inhibitory protein being immune regulators. miRNA‐33‐c had seven putative targets with KO annotations, with only E3 ubiquitin‐protein ligase HECW2 linked to immunity. Further discussion will focus on TRAF4 and TRAF6, as their function has been studied in cnidarians (Girosi et al. 2007; Pierobon et al. 2004; Quistad et al. 2014; Steichele et al. 2021), while E3 ubiquitin‐protein ligase synoviolin, E3 ubiquitin‐protein ligase HECW2, GABARAP, and MAP3K12 are immune‐related but have only been verified in mammals to date (Choi et al. 2016; Kim et al. 2018; Wan et al. 2021; Yagishita et al. 2008).

**TABLE 4:** Significantly differentially expressed miRNAs for main effect time, exposure, and resistance, along with the interactions.

miRNA	Type	Main effect	NumDF	DenDF	F‐value	*p*	FDR *p*‐value
miRNA_33‐c	Acroporid	**Time**	**1**	**100.961**	**32.716**	**< 0.0001**	**< 0.0001**
		Exposure	1	12.793	0.048	0.829	0.981
		**Resistance**	**1**	**45.225**	**17.391**	**0.00014**	**0.0046**
		Time: Exposure	1	100.961	2.586	0.111	0.955
		Time: Resistance	1	100.855	2.675	0.105	0.391
		Exposure: Resistance	1	45.225	0.677	0.415	0.999
		Time: Exposure: Resistance	1	100.855	0.29	0.591	0.977
miRNA_2‐3p‐c	Acroporid	**Time**	**1**	**564.552**	**20.766**	**< 0.0001**	**< 0.0001**
		Exposure	1	90.048	0.556	0.458	0.981
		Resistance	1	96.898	0.792	0.376	0.899
		Time: Exposure	1	565.227	0.059	0.808	0.980
		**Time: Resistance**	**1**	**564.631**	**24.887**	**< 0.0001**	**< 0.0001**
		Exposure: Resistance	1	252.722	0.400	0.528	0.999
		Time: Exposure: Resistance	1	565.362	0.054	0.817	0.977
miRNA_2022	Cnidarian	**Time**	**1**	**172.169**	**8.118**	**0.005**	**0.0087**
		Exposure	1	27.929	0.434	0.515	0.981
		**Resistance**	**1**	**61.126**	**26.405**	**< 0.0001**	**0.0002**
		Time: Exposure	1	172.283	4.613	0.033	0.705
		**Time: Resistance**	**1**	**172.298**	**11.546**	**0.0008**	**0.02**
		Exposure: Resistance	1	78.559	0.037	0.847	0.999
		Time: Exposure: Resistance	1	172.452	0.0346	0.853	0.977

*Note:* Significant *p*‐values marked in bold.

Abbreviations: DenDF, denominator degrees of freedom; FDR, false discovery rate; F‐value, F statistic; NumDF, numerator degrees of freedom.

**FIGURE 4:**
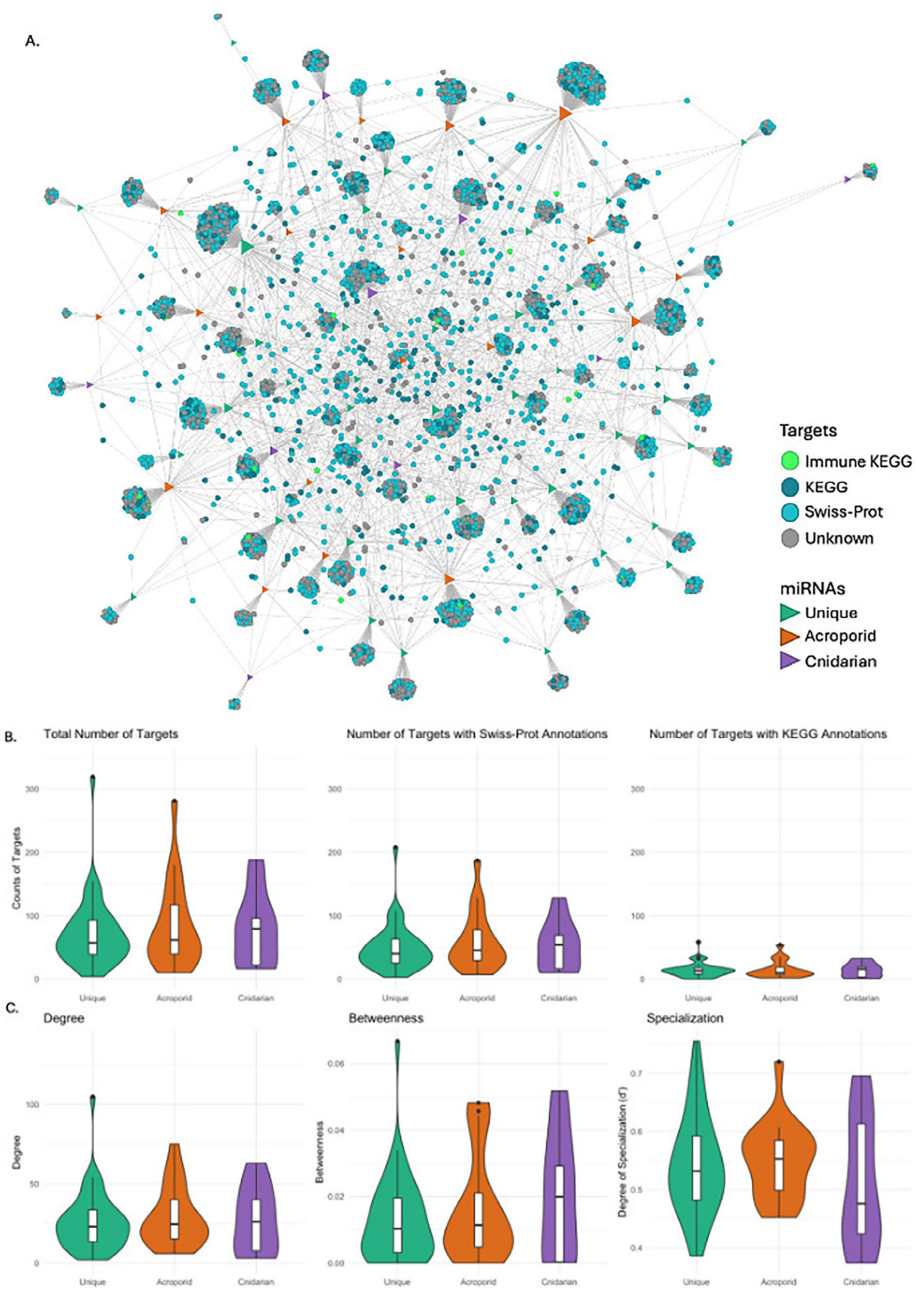
(A) Bipartite (two‐mode) network showing *A. cervicornis* miRNAs and their extended 13‐seed targets. Nodes are colored by either miRNA class or annotation type. miRNAs are triangles, while their respective targets are circles. miRNA node size is proportional to their degree (larger nodes are equal to more connections). (B) miRNA target counts for extended 13‐seed miRNA: MRNA pairing across unique, acroporid, and cnidarian classes for all gene targets, Swiss‐Prot Annotations, KEGG orthology (KO) annotations (left to right). (C) miRNA centrality measures degree, betweenness, and degree of specialization (from left to right) for miRNAs within each miRNA class.

miRNA–mRNA target analyses first looked at what proportion of predicted miRNA targets with 13‐seed extended targets were in CDS compared to 3′ UTR sites and across each miRNA class (i.e., unique, acroporid, and cnidarian). Out of 28,059 validated genes in *A. cervicornis* , there were 3946 CDS target locations contained within 3355 unique genes, which was a significantly higher proportion than the 1097 3′ UTR target locations out of 11,168 UTR locations (*χ* ^2^_(1)_ = 35.945, *p* ≤ 0.0001). Only three genes contained miRNA targets in both a CDS and 3′ UTR regions, which were F‐type H + ‐transporting ATPase subunit alpha, sarcosine dehydrogenase, and another with no KO annotation but with a Swiss‐Prot annotation for GATOR complex protein, none of which have links to immune regulation in Cnidaria (Fillingame et al. 2000; Oka et al. 1979; Wei et al. 2019). There is an average of 76.6 ± 7.2 SE total extended 13‐seed predicted mRNA targets per miRNA (52.8 ± 4.8 SE had Swiss‐Prot annotations and 14.9 ± 1.5 SE had KO annotations), and there is no significant differences in log‐transformed target counts between miRNA classes (i.e., cnidarian, acroporid, unique; total targets: *F*_2,64_ = 0.528, *p* = 0.593; Swiss‐Prot targets: *F*_2,64_ = 0.477, *p* = 0.623; KO targets: *F*_2,64_ = 0.277, *p* = 0.759; Table 5 and Figure 4B). Looking further into plant miRNA characteristics compared to cnidarian miRNAs, of the extended 13‐seed putative targets, ~20% of miRNAs had complementarity matches to the mRNA targets of ≥ 15 miRNA nucleotides. In addition, there is a 33% more chance that a single miRNA will target CDS regions compared to a 3′ UTR region (*χ* ^2^_(1)_ = 59.266, *p* ≤ 0.0001). There was no difference in the mean number of CDS hits (*F*_2,64_ = 0.006, *p* = 0.994) or 3′ UTR hits (*F*_2,64_ = 0.188, *p* = 0.829) between miRNA classes (i.e., cnidarian, acroporid, unique).

**TABLE 5:** Mean number of *A. cervicornis* miRNA targets ± SE and mean numbers with each annotation type (either Swiss‐Prot or KEGG Orthology) ± SE separated into unique *A. cervicornis* miRNAs, shared with other acroporid miRNAs, and shared with other cnidarian miRNA classes, as well as the average across all groups. Numbers in parentheses correspond to the number of miRNAs in each class. Pre‐filter is the full 7‐seed pita targets, and post‐filter is the extended 13‐seed pita targets.

miRNA class	Annotation type					
	Pre‐filter			Post‐filter		
	Raw ± SE	Swiss‐Prot ± SE	KO ± SE	Raw ± SE	Swiss‐Prot ± SE	KO ± SE
Unique (39)	686.9 ± 120.7	473.3 ± 83.4	141.3 ± 25	72.6 ± 8.7	49.6 ± 5.9	14.6 ± 1.8
Acroporid (18)	914.8 ± 216.8	624.9 ± 147.3	177.8 ± 42.2	84.7 ± 16.4	59.8 ± 11.0	15.8 ± 3.3
Cnidarian (10)	782.5 ± 167.4	522.7 ± 113.3	150.3 ± 33	77.5 ± 18.7	52.7 ± 12.6	14.4 ± 3.6
Total (67)	762.4 ± 94.1	521.4 ± 64.5	152.4 ± 18.9	76.6 ± 7.2	52.8 ± 4.8	14.9 ± 1.5

Abbreviations: KO, KEGG orthology; SE, standard error.

The bipartite network consisted of 67 miRNAs with 4893 predicted targets, 1548 having unknown annotations, 2397 having Swiss‐Prot annotations, and 948 having KO annotations, 56 of the KO annotations having a minor category of “Immune system” (Figure 4A). We calculated three centrality measures—degree, betweenness, and degree of specialization (d')—where the degree is the number of targets a miRNA has; betweenness calculates the fewest number of targets it takes to connect to another miRNA, and d' measures the amount to which a miRNA's targets are connected to other miRNAs, where 1 is a perfect specialist (a miRNA and its targets are completely disconnected from the other miRNAs and their targets). There is no significant difference in degree (*F*_2,64_ = 0.414, *p* = 0.663), betweenness (*F*_2,64_ = 0.266, *p* = 0.768), or d' (*F*_2,64_ = 1.08, *p* = 0.346) between miRNA classes (Figure 4C) and this did not change if seed match was increased. Cnidarian miRNAs overall tend to have the lowest median d' and highest median betweenness scores, while the median degree across miRNA classes is almost the same, ranging from 23 to 26 (Figure 4C).

Community detection of our unweighted bipartite network output 16 different modules of miRNAs and their predicted mRNA targets with a modularity score of 0.468 where anything above 0.3 is considered a good community structure (Clauset et al. 2004). Good community structure indicates miRNAs are clustering together to target similar transcripts. Ten of the 16 modules had significantly over‐represented pathways according to Fisher's exact tests (Table 6). The significantly over‐represented pathways came from 23 different KEGG genes at 36 locations (27 CDS and 9 3′ UTR). Module 8 was over‐represented by pathways involved in coral immunity and was made up of two unique *A. cervicornis* miRNAs (Acerv_scaffold_16_9855 and Acerv_scaffold_3_2245). When considering targets of higher seed match (i.e., 14+ or 15+ etc.) there was no significant over‐representation of immune pathways, indicating that this immune module was created from putative targets with a 13‐seed match. Focusing on immune regulators, caspase 7 was the KEGG gene responsible for the over‐representation of the apoptosis pathway (map04210, *p* < 0.01), and TRAF6 was the KEGG gene driving over‐representation in both endocytosis (map04144, *p* < 0.01) and Toll‐like receptor signaling pathway (map04620, *p* < 0.01). The notch signaling pathway (map04330, *p* < 0.01) was also over‐represented in this module, which is hypothesized to be involved in coral neurogenesis and tissue regeneration but has not been functionally explored in any coral to date (Lv et al. 2024). Interestingly, two of the three miRNAs differentially expressed due to disease resistance, miRNA‐2022 and miRNA_2‐3p, are in modules with pathways that are significantly over‐represented and the genes responsible for the over‐representation are some of the same immune regulators highlighted in our differential expression analysis. miRNA‐2022 contributes to the over‐representation of a protein processing pathway (map04141, *p* < 0.01) via E3 ubiquitin‐protein ligase synoviolin in module 1 and miRNA_2‐3p contributes to the over‐representation of mitophagy (map04137, *p* < 0.01) and autophagy (map04140, *p* < 0.001) pathways via GABARAP in module 13 (Table 6).

**TABLE 6:** miRNAs and their associated modules from community detection where significantly over‐represented pathways are listed. Includes module number and miRNAs, the significant KEGG pathway with FDR adjusted *p*‐value in parentheses, the KEGG gene within that pathway responsible for significant effect, and location(s) in the *A. cervicornis* genome and if they are in CDS or 3' UTR regions.

Module	microRNAs	Significant pathways (FDR adj. *p*‐value)	KEGG genes	Location (CDS or UTR)
1	Acerv_scaffold_151_44408; Acerv_scaffold_51_24751; **miRNA_2022;** miRNA_2050	Estrogen signaling pathway (0.00507)	Gamma‐aminobutyric acid type B receptor	Acerv_scaffold_1:3444643–3445685 (UTR)
			Apoptosis regulator Bcl‐2	Acerv_scaffold_81:485286–492951 (UTR)
			Gamma‐aminobutyric acid type B receptor	Acerv_scaffold_1:3162151–3162327 (CDS); Acerv_scaffold_53:991605–991856 (CDS)
		Protein processing in endoplasmic reticulum (0.00507)	Peptide‐N4‐(N‐acetyl‐beta‐glucosaminyl) asparagine—amidase	Acerv_scaffold_20:2443389–2445603 (CDS)
			Apoptosis regulator Bcl‐2	Acerv_scaffold_81:485286–492951 (UTR)
			E3 ubiquitin‐protein ligase synoviolin	Acerv_scaffold_13:330469–332482 (UTR)
2	Acerv_scaffold_15_9107; Acerv_scaffold_4_2537; Acerv_scaffold_72_32054; miRNA_2‐c; miRNA_2025; miRNA_24‐3p‐c; miRNA_9425	Regulation of lipolysis in adipocytes (0.00209)	Adrenergic receptor beta‐2	Acerv_scaffold_11:3584545–3585648 (CDS); Acerv_scaffold_69:432898–433917 (CDS); Acerv_scaffold_69:445941–446960 (CDS)
		cGMP‐PKG signaling pathway (0.0418)	Adrenergic receptor beta‐2	Acerv_scaffold_11:3584545–3585648 (CDS); Acerv_scaffold_69:432898–433917 (CDS); Acerv_scaffold_69:445941–446960 (CDS)
		Hippo signaling pathway (0.00209)	Protein scribble	Acerv_scaffold_36:592177–593843 (UTR)
			Wingless‐type MMTV integration site family, member 8	Acerv_scaffold_36:448667–448935 (CDS)
3	Acerv_scaffold_16_9509; miRNA_100; miRNA_2036; miRNA_5‐3p‐c	Fatty acid elongation (0.00723)	Elongation of very long chain fatty acids protein 4	Acerv_scaffold_18:1852442–1853251 (CDS)
		Salivary secretion (0.0156)	Deleted in malignant brain tumors 1 protein	Acerv_scaffold_26:2479226–2479498 (CDS)
		Vitamin digestion and absorption (0.0465)	ATP‐binding cassette, subfamily C (CFTR/MRP), member 1	Acerv_scaffold_97:155020–155160 (CDS)
		Fatty acid metabolism (0.00014); Biosynthesis of unsaturated fatty acids (0.00014)	Elongation of very long chain fatty acids protein 4	Acerv_scaffold_18:1852442–1853251 (CDS)
			(3R)‐3‐hydroxyacyl‐CoA dehydrogenase	Acerv_scaffold_0:3975108–3976247 (CDS)
		Cell cycle (0.00723); Meiosis (0.00723); Oocyte meiosis (0.00723), Progesterone‐mediated oocyte maturation (0.00723)	Anaphase‐promoting complex subunit 7	Acerv_scaffold_0:1131575–1131704 (CDS)
4	Acerv_scaffold_112_40019; Acerv_scaffold_84_34634	None		
5	Acerv_scaffold_23_14072; Acerv_scaffold_23_14118; Acerv_scaffold_38_19930; Acerv_scaffold_57_27039; Acerv_scaffold_64_30361	Signaling pathways regulating pluripotency of stem cells (0.000404); MAPK signaling pathway (0.000139); Ras signaling pathway (0.000404); Rap1 signaling pathway (0.000404); PI3K‐Akt signaling pathway (0.00195); Adherens junction (0.000000537); Thermogenesis (0.000000537); Regulation of Actin cytoskeleton (0.000000537); Parathyroid hormone synthesis, secretion and action (0.000693)	Fibroblast growth factor receptor 1	Acerv_scaffold_18:196199–196351 (UTR); Acerv_scaffold_5:5488471–5488746 (CDS)
6	Acerv_scaffold_32_17956; Acerv_scaffold_9_6292; miRNA_4‐3p‐c; miRNA_7‐3p‐c	Inositol phosphate metabolism (0.0448); Chemokine signaling pathway (0.0448); Phosphatidylinositol signaling system (0.0448); Vascular smooth muscle contraction (0.0448); Apelin signaling pathway (0.0448); Platelet activation; Neutrophil extracellular trap formation (0.0448); Retrograde endocannabinoid signaling (0.0448); Long‐term depression; Phototransduction (0.0448); Inflammatory mediator regulation of TRP channels (0.0448); Insulin secretion; GnRH signaling pathway (0.0448); Thyroid hormone synthesis (0.0448); Relaxin signaling pathway (0.0448); Growth hormone synthesis, secretion and action (0.0448); Endocrine and other factor‐regulated calcium reabsorption (0.0448); Carbohydrate digestion and absorption (0.0448)	Phosphatidylinositol Phospholipase C, beta	Acerv_scaffold_35:931600–935804 (CDS)
7	Acerv_scaffold_54_26073; Acerv_scaffold_79_33834; miRNA_14‐c	Mucin type O‐glycan biosynthesis (0.029); Other types of O‐glycan biosynthesis	Polypeptide N‐acetylgalactosaminyl transferase	Acerv_scaffold_10:3523857–3523967 (CDS)
		Ovarian steroidogenesis (0.029); Fat digestion and absorption (0.029); Cholesterol metabolism (0.029)	Scavenger receptor class B, member 1	Acerv_scaffold_81:673130–674686 (CDS)
		Spliceosome (0.029)	Nuclear cap‐binding protein subunit 1	Acerv_scaffold_21:859881–859991 (CDS)
		RNA polymerase (0.029)	DNA‐directed RNA polymerase I subunit RPA2	Acerv_scaffold_7:2694968–2695027 (CDS)
8	Acerv_scaffold_16_9855; Acerv_scaffold_3_2245	Apoptosis (0.00183)	Caspase 7	Acerv_scaffold_11:1937977–1938257 (CDS)
		Endocytosis (0.00183); Toll‐like receptor signaling pathway (0.00183); IL‐17 signaling pathway (0.012); Osteoclast differentiation (0.0232); MAPK signaling pathway (0.012); NF‐kappa B signaling pathway (0.0391); RIG‐I‐like receptor signaling pathway (0.012); Neurotrophin signaling pathway (0.012)	TNF receptor‐associated factor 6	Acerv_scaffold_10:295843–30494 (UTR)
		Notch signaling pathway (0.00183)	Deltex	Acerv_scaffold_69:295948–296215 (CDS); Acerv_scaffold_271:591–858 (CDS)
9	Acerv_scaffold_26_14789; miRNA_29‐c; miRNA_5‐c	Metabolic pathways (0.00180); Various types of N‐glycan biosynthesis (0.000000201)	Beta‐1,4‐N‐acetylgalactosaminyl transferase 4	Acerv_scaffold_11:4917323–4918228 (CDS); Acerv_scaffold_16:116390–117469 (CDS)
10	Acerv_scaffold_116_40356; Acerv_scaffold_14_8906; Acerv_scaffold_159_44966; Acerv_scaffold_2_1277; Acerv_scaffold_36_19235; Acerv_scaffold_57_26966; Acerv_scaffold_8_5139; miRNA_7‐c	PI3K‐Akt signaling pathway (0.00354); Focal adhesion (0.0000155); ECM‐receptor interaction (0.000000966)	Tenascin	Acerv_scaffold_4:2128008–2130294 (CDS); Acerv_scaffold_4:2144285–2147661 (CDS)
		Fc epsilon RI signaling pathway (0.0103); Fc gamma R‐mediated phagocytosis (0.0103)	GRB2‐associated‐binding protein 2	Acerv_scaffold_98:540186–541964 (CDS)
11	Acerv_scaffold_0_318; Acerv_scaffold_28_16363; Acerv_scaffold_50_24648; miRNA_13‐c; miRNA_17‐3p‐c; miRNA_2023; miRNA_2030; miRNA_3‐3p‐c; miRNA_4‐c	Wnt signaling pathway (0.0182)	Catenin delta‐2	Acerv_scaffold_11:4682459–4682642 (CDS)
		Thyroid hormone signaling pathway (0.0182)	Thyroxine 5‐deiodinase	Acerv_scaffold_17:1341880–1347709 (UTR); Acerv_scaffold_34:536366–538377 (UTR)
		DNA replication (0.0215); Nucleotide excision repair (0.0215)	DNA polymerase epsilon subunit 1	Acerv_scaffold_1:2891316–2894545 (UTR)
12	Acerv_scaffold_33_18099; miRNA_19‐c; miRNA_2037	Neuroactive ligand‐receptor interaction (0.000000214)	Histamine receptor H2	Acerv_scaffold_75:353325–353890 (CDS); Acerv_scaffold_4:5218138–5218174 (CDS)
			Neuropeptide FF receptor 2	Acerv_scaffold_93:201956–202338 (CDS)
			Pyroglutamylated RFamide peptide receptor	Acerv_scaffold_0:445655–445874 (CDS); Acerv_scaffold_6:4632024–4632502 (UTR)
		Gastric acid secretion (0.00498)	Histamine receptor H2	Acerv_scaffold_75:353325–353890 (CDS); Acerv_scaffold_4:5218138–5218174 (CDS)
13	miRNA_1‐3p‐c; **miRNA_2‐3p‐c**	Synaptic vesicle cycle (0.000236); Dopaminergic synapse (0.00191)	Solute carrier family 6	Acerv_scaffold_0:2724629–2725015 (UTR)
		Mitophagy (0.0012); Autophagy (0.000297); Longevity regulating pathway (0.0285); FoxO signaling pathway (0.0285)	GABA(A) receptor‐associated protein	Acerv_scaffold_80:1108563–1109392 (CDS)
		Ribosome biogenesis in eukaryotes (0.0285)	Periodic tryptophan protein 2	Acerv_scaffold_174:68391–69025 (CDS)
14	Acerv_scaffold_17_30; Acerv_scaffold_20_11778; Acerv_scaffold_23_14008; Acerv_scaffold_33_18101; Acerv_scaffold_3_1884; Acerv_scaffold_48_24090; miRNA_8‐3p‐c	None		
15	Acerv_scaffold_20_11433	Motor proteins (0.00152)	Centromeric protein E	Acerv_scaffold_73:534469–534546 (CDS)
		Protein digestion and Absorption (< 0.0001)	Collagen type XII alpha	Acerv_scaffold_0:2542620–2543087 (CDS)
16	Acerv_scaffold_120_41029; miRNA_10‐c; **miRNA_33‐c**	Retinol metabolism (0.0365)	All‐trans‐retinol 3, 4‐desaturase	Acerv_scaffold_5:2337635–2338499 (UTR)

*Note:* miRNAs were significantly differentially expressed for either resistance or the interaction between time and resistance marked in bold.

Abbreviations: CDS, coding domain sequence; UTR, untranslated region.

## 4 Discussion

Our small RNA sequencing identified 67 *bona fide Acropora cervicornis* miRNAs categorized into three miRNA classes—unique, acroporid, and cnidarian. *A. cervicornis* had 39 unique miRNAs, 18 shared acroporid miRNAs, and 10 conserved cnidarian miRNAs. The large number of unique miRNAs in *A. cervicornis* , compared to the other *Acropora* species ( *A. millepora* —20 and *A. digitifera* —13) is likely driven by the depth of sequencing across genotypes. Our data with ~385 million reads was 12× and 25× greater sequencing depth than the studies sequencing *A. millepora* (Praher et al. 2021) and *A. digitifera* (Gajigan and Conaco 2017) and greater sequencing depth has been shown to increase miRNA diversity (Khamina et al. 2022). Our updated phylogenetic comparison of miRNAs across the cnidarians confirms that there is one shared bilaterian and two universal cnidarian miRNAs, but indicates that there is now only one conserved anthozoan miRNA, with the remaining five miRNAs being conserved across Hexacorallia. *Acropora* corals had the highest number of conserved miRNAs across the cnidarian phylogenetic tree, with 18 being conserved in at least two of the three *Acropora* species in our study.

Network analyses of *A. cervicornis* miRNA–mRNA interactions indicate that *A. cervicornis* miRNAs have a similar number of putative targets and centrality measures regardless of miRNA class (i.e., unique, acroporid, cnidarian) and significantly more putative targets in CDS regions than 3′ UTR, suggesting they operate more similarly to plants in their target regulation (Chung et al. 2017; Reinhart et al. 2002). Community detection of our filtered miRNA–mRNA target network found 16 different modules, 10 of which had significantly over‐represented KEGG pathways, with one module containing gene types, TNFs, and caspases, previously induced by WBD exposure in *A. cervicornis* (Libro and Vollmer 2016). Interestingly, the differential expression of miRNAs during disease exposure was primarily confined to temporal effects, with only three miRNAs being differentially expressed due to the disease resistance of the coral genotypes, and no miRNA being differentially expressed due to disease exposure. Nevertheless, all the differentially expressed miRNAs associated with disease resistance had putative mRNA targets of immune‐related genes, including those highlighted in our over‐representation analysis of our miRNA modules.

### 4.1 Phylogenetic Conservation of Cnidarian miRNAs

In addition to identifying 67 *bona fide* *A. cervicornis* miRNAs, our phylogenetic comparisons of cnidarian miRNAs considered an additional 16 miRNA datasets from a solitary hydrozoan, three scyphozoans, an octocoral, four stony corals, and seven anemones, and built on the previous survey of cnidarian miRNAs by Praher et al. (2021) that examined 10 species. The addition of the three scyphozoan miRNA repertoires shows the same miRNA conservation as the single hydrozoan in our study, *Hydra vulgaris*, with the presence of miRNA‐2022 and miRNA‐2030 and loss of miRNA‐100 (Krishna et al. 2013; Nong et al. 2020). Interestingly, the addition of the octocoral *Heliopora coerulea* also shows a loss of bilaterian miRNA‐100 and only two conserved miRNAs overall, upending the previously suggested higher miRNA conservation in anthozoans and reducing the set of conserved anthozoan miRNAs from six miRNAs (Praher et al. 2021) to one miRNA (miRNA‐2023). Thus, as more new taxa of cnidarians are added, we uncover less miRNA conservation across cnidarians and see much more specialization within the cnidarian classes in the phylum.

The scleractinian corals in our study included two Indo‐Pacific *Acropora* species, one Caribbean *Acropora* species, *Cataphyllia jardinei*, and *Stylophora pistillata* , which have 10 miRNAs in common with only one shared within Scleractinia exclusively (miRNA‐14). No miRNAs are shared only between *C. jardinei* and *S. pistillata* ; however, the *Acropora* species are distinctive because the three *Acropora* share 18 miRNAs, predating the divergence of the Indo‐Pacific and Caribbean clades about 58–68 million years ago (mya) (Selwyn and Vollmer 2023; Wallace 1999). We could postulate several potential reasons why there may be an increased number of conserved miRNAs in the Acroporidae family. First, it could simply be due to the fact that there were many more *Acropora* genomes analyzed in our study compared to the other cnidarian species, allowing us to uncover more conserved small RNAs. Second, the *Acropora* species in our study diverged from each other much later than the other cnidarian species, such as the anemones which split from stony corals about over 300 mya (Selwyn and Vollmer 2023) and contain far less similar genomes to each other suggesting longer divergence times (Spano et al. 2018). Additionally, it could be due to acroporids increased organismal complexity. They have the most complex polyp‐canal system in all Scleractinia (Li et al. 2023), and miRNAs have been postulated as developmental and evolutionary determinants of organismal complexity in vertebrates (Heimberg et al. 2008). Lastly, the clear‐cut contrast between the conservation of miRNAs within Scleractinia could also be a distinction between robust and complex coral clades. However, with three complex corals (all acroporids) and only one definitive robust coral ( *S. pistillata* ; Voolstra et al. 2017) accounted for in our study, and one unknown clade classification ( *C. jardinei* ; Fukami et al. 2008), more taxa would need to be sampled for this hypothesis to be validated.

### 4.2 miRNA Target Analysis in *A. cervicornis*

miRNAs are an essential part of complex regulatory networks that control various cellular processes (Liu et al. 2009; Zhou et al. 2011), including innate immune responses (Gracias and Katsikis 2011) that aid pathogen clearance and ensure a rapid return to homeostasis (Nejad et al. 2018). Our miRNA target analysis focused on highlighting the putative targets of the three differentially expressed miRNAs potentially aiding in the survival of disease resistance genotypes and conducting an over‐representation analysis of KEGG pathways on the target network modules to distinguish miRNAs working together in an immune‐related context. Innate immune responses are tightly regulated to rapidly clear infection while avoiding excessive stress response and protecting the host (Momen‐Heravi and Bala 2018) whereby miRNAs, in their ability to down‐regulate targets at multiple levels along a signaling cascade (Gantier et al. 2012; Nejad et al. 2018) can help maintain this immune homeostasis in the presence of environmental stressors such as disease.

When considering the trends of the differentially expressed miRNAs, it is important to consider our experimental design. All tanks were dosed with coral tissue, the difference being that disease tanks contained WBD‐associated bacteria while the healthy tanks theoretically did not, but this could be a potential reason for the lack of differential expression signature in our exposure treatment. Cnidarians containing only an innate immune system apply a generalized immune response (Bosch and Rosenstiel 2015), and therefore, the coral host may have elicited an immune response to its own tissue or even the commensal microbes present in the healthy tanks, attributing to no differential expression between the healthy and diseased tanks. In contrast, the resistance signature comes from a resistant genotype inherent to the organism itself, and these resistant individuals are responding differently over time in three miRNAs. These three miRNAs differentially expressed due to resistance may be aiding in the regulation of the coral immune system in the presence of coral tissue, and both commensal and pathogenic microbes in our study.

All three differentially expressed miRNAs were conserved in the *Acropora* species, but only miRNA‐2022 has predicted targets involved in a host innate immune system response to infection in Cnidaria. Prior work indicates that miRNA‐2022 is a key miRNA in cnidarians involved in symbioses and cnidocyte formation. miRNA‐2022 was up‐regulated upon symbiont infection in the anemone, *Exaiptasia pallida*, targeting a downstream intracellular messenger of the fibroblast growth factor signaling cascade (Baumgarten et al. 2018). miRNA‐2022 was also shown to be specifically expressed in the stinging cells (cnidocytes) of the anemone, *Nematostella vectensis* , and hydroid, *Hydractinia symbiolongicarpus* , and miRNA‐2022 knock‐down experiments prevented the creation of the stinging capsule (Fridrich et al. 2023). Furthermore, cnidocytes in *N. vectensis* were demonstrated to affect the expression of a specific Toll‐like receptor (TLR) that activates the downstream NF‐kappa B signaling pathway, suggesting a potential link between miRNA‐2022 and the expression of a prominent innate immune pathway in Cnidaria (Brennan et al. 2017).

Within *A. cervicornis* , miRNA‐2022 putatively regulates multiple forms of E3 ubiquitin‐protein ligases—TNF receptor‐associated factor four (TRAF4) and six (TRAF6) (Jiang and Chen 2012). miRNA‐2‐3p‐c and miRNA‐33‐c both have predicted immune‐related targets but neither has been validated in coral immunity to date (Kim et al. 2018; Wan et al. 2021). E3 ubiquitin enzymes are mainly responsible for recognizing protein substrates for degradation or modifying protein–protein interactions (Hu and Sun 2016). In response to a pathogen, the host innate immune system launches an array of distinct antimicrobial activities such as inflammatory signaling cascades, autophagy, and apoptosis, all of which can be fine‐tuned by the ubiquitin system to eradicate the invading pathogens and reduce host damage (Li et al. 2016). Additionally, TRAF4 and TRAF6 are both involved in the TLR to NF‐kappa B signaling pathway (Seneca et al. 2020), providing another link in this specific miRNA's potential role in regulating an immune pathway in Cnidaria, this time in a stony coral.

Our over‐representation analysis also highlighted the importance of these TRAF family of proteins along with caspases as the putative targets of two unique *A. cervicornis* miRNAs. TRAFs are important regulators of the apoptosis cascade that initiate signal transduction pathways and result in caspase activation and apoptosis (Bi et al. 2009). Apoptosis is an important component of organismal responses to stress and pathogenic infection (Fulda et al. 2010). Initially, controlled apoptosis of infected cells may serve to prevent further infection of an organism (Man and Kanneganti 2016); however, persistent and increased apoptosis may contribute to organismal death (Ainsworth et al. 2007; Liu et al. 2005). Prior literature shows that TRAFs and caspases are up‐regulated in WBD‐infected *A. cervicornis* (Libro et al. 2013); therefore, miRNAs ability to inhibit transcription of apoptosis regulators via targeting TRAF6 and caspase 7 could ultimately prove beneficial for *A. cervicornis* infected with WBD to prevent over‐expression and maintain homeostasis. Lastly, TRAFs are involved in regulating the immune system through the activation of the nuclear factor kappa B (NF‐kappa B) (Zhou et al. 2011) which is known to promote the transcription of many miRNAs in mammals (Nejad et al. 2018). This feedback of miRNAs on innate immune pathways may fine‐tune the coral's response to infection and protect the coral host.

Overall, our data highlight unique characteristics of conserved miRNAs and update the distribution of miRNAs along the cnidarian phylogenetic tree, where increasing the number of species resulted in less conservation at higher taxonomic levels and a greater number of species‐specific miRNAs. *Acropora* corals show an apparent increase in their small RNA repertoire relative to other corals, which warrants further investigation. There were three differentially abundant miRNAs in resistant coral genotypes, which had putative targets related to innate immunity and focused around signaling cascades. Our network and over‐representation analysis highlighted key genes such as TNF receptor‐associated factor 6 and caspase 7 that are involved in regulating multiple important immune‐related pathways as predicted targets of several unique *A. cervicornis* miRNAs. Together, these miRNAs may help clear pathogenic infection, but more so, the miRNA repertoire in *A. cervicornis* and their vast number of putative targets more likely aid in the maintenance of immune homeostasis in the presence of environmental stress such as disease infection.

## Author Contributions

**Brecia A. Despard:** data curation (equal), formal analysis (equal), investigation (equal), methodology (equal), visualization (equal), writing – original draft (lead), writing – review and editing (equal). **Jason D. Selwyn:** formal analysis (equal), investigation (equal), methodology (equal), writing – review and editing (equal). **Allison N. Shupp:** data curation (equal), writing – review and editing (equal). **Steven V. Vollmer:** conceptualization (equal), data curation (equal), funding acquisition (equal), investigation (equal), methodology (equal), project administration (equal), writing – review and editing (equal).

## Conflicts of Interest

The authors declare no conflicts of interest.

## Data Availability

Small RNA and mRNA sequencing data used for microRNA and target discovery can be accessed from NCBI BioProject PRJNA1187871 and NCBI BioProject PRJNA949884 (respectively). microRNA discovery and target analysis code are available from GitHub: https://github.com/VollmerLab/miRNA_Characterization.
